# Rush Medical College

**Published:** 1845-10

**Authors:** 


### RUSH MEDICAL COLLEGE.

From the annual circular of the Rush Medical College we learn that William B. Herrick, M.D., favorably known in this city as a laborious and minute anatomist, has received the appointment of Professor of General and Descriptive Anatomy in that institution. The selection, we have reason to believe, will be satisfactory to the friends of the school. If Dr. Herrick's powers as a lecturer should prove to be equal to his industry and attainments as an anatomist, he must take a high rank among western teachers. The cordial wishes of his Louisville friends attend him in his new career. The lectures in this College commence on the first Monday in November and continue 16 weeks. Y.

